# Selection of Reference Genes for qPCR Analyses of Gene Expression in Ramie Leaves and Roots across Eleven Abiotic/Biotic Treatments

**DOI:** 10.1038/s41598-019-56640-3

**Published:** 2019-12-27

**Authors:** Yongting Yu, Gang Zhang, Yikun Chen, Qingqing Bai, Chunsheng Gao, Liangbin Zeng, Zhimin Li, Yi Cheng, Jia Chen, Xiangping Sun, Litao Guo, Jianping Xu, Zhun Yan

**Affiliations:** 1 Department of Plant Protection, Institute of Bast Fiber Crops and Center for Southern Economic Crops, Chinese Academy of Agricultural Science, Changsha, 410205 China; 2 College of Pharmacy, Shaanxi University of Chinese Medicine, Xianyang, 712406 China; 3 Department of Biology, McMaster University, Hamilton, Ontario L8S 4K1 Canada

**Keywords:** Gene expression analysis, Plant molecular biology

## Abstract

Quantitative real-time PCR (qPCR) is commonly used for deciphering gene functions. For effective qPCR analyses, suitable reference genes are needed for normalization. The objective of this study is to identify the appropriate reference gene(s) for qPCR analyses of the leaves and roots of ramie (*Boehmeria nivea* L.), an important natural fiber crop. To accomplish this goal, we investigated the expression patterns of eight common plant qPCR reference genes in ramie leaves and roots under five abiotic stresses, five hormonal treatments, and one biotic stress. The relative expression stabilities of the eight genes were evaluated using four common but different approaches: geNorm, NormFinder, BestKeeper, and RefFinder. Across the 11 tested conditions, *ACT1* was the most stably expressed among the eight genes while *GAPDH* displayed the biggest variation. Overall, while variations in the suggested reference genes were found for different tissue x treatment combinations, our analyses revealed that together, genes *ACT1*, *CYP2*, and *UBQ* can provide robust references for gene expression studies of ramie leaves under most conditions, while genes *EF-1α*, *TUB*, and *ACT1* can be used for similar studies of ramie roots. Our results should help future functional studies of the genes in ramie genome across tissues and environmental conditions.

## Introduction

RNA abundance analysis is an important approach for studying gene functions. Frequently used methods for quantifying RNA abundance include Northern blotting^1^, ribonuclease protection assay (RPA)^2^, semi-quantitative reverse transcription polymerase chain reaction (sRT-PCR)^3^, and quantitative real-time PCR (qPCR)^4^. Among these methods, qPCR has become an increasingly prevalent method due to its high specificity, high sensitivity, wide dynamic range, and relatively low cost. qPCR experiment is often used to validate gene expression data obtained from microarray hybridization or from transcriptomics based on next-generation sequencing platforms. However, the usefulness and interpretation of qPCR results depend heavily on a number of factors, including the quality and quantity of extracted RNA samples, the efficiency of the reverse transcription reaction, random errors in experiments, and the reference gene selected for normalization and comparison^5–7^. Among these factors, the expression stability of the reference gene is crucial for minimizing systematic biases and for accurately normalizing target gene expression using qPCR. Therefore, identifying suitable and reliable internal reference genes represents a critical step in qPCR analyses.

Many studies have been conducted to identify reference genes for qPCR in different organisms, targeting different tissues, developmental stages, and environmental conditions. For plants, a number of genes have been frequently used as reference genes for qPCR, including the following house-keeping genes: *18S rRNA*, *ACT* (β or γ actin), *TUB* (α or β tubulin), *EF-1*α (elongation factor 1α), *GAPDH* (glyceraldehyde-3-phosphate dehydrogenase), and *UBQ* (poly-ubiquitin)^8–12^. In addition, several new genes were recently identified as stably expressed in different plants and plant tissues under selected conditions. These new candidate reference genes include *F-box* (a F-box family protein), *SAND* (a SAND family protein), *PP2A* (protein phosphatase 2 A), *PEPKR1* (phosphoenolpyruvate carboxylase-related kinase 1), *TIP41* (Tap42-interacting protein of 41 kDa), *eIF-4α* (eukaryotic translation initiation factor 4α), *CRKs* (CDPK-related kinases), *ABC* (ATP-binding cassette transporter), *CYP2* (cyclophilin2), *ELF1* (eukaryotic elongation factor 1, α or β), *IDE* (insulin-degrading enzyme), *CBP20* (carotenoid-binding protein 20), *UBC* (ubiquitin-conjugating enzyme), and *SamDC* (S-adenosylmethionine decarboxylase gene)^8–11^.

Ramie (*Boehmeria nivea* L.) or “China grass” is an important natural fiber crop mainly grown in China, India, and Southeast Asian and other Pacific Rim countries^12^. Aside from its long stem fiber, other components of the plant are also of significant economic value, including a high level of crude protein contents in leaves and shoots that make them an ideal source of feed for beef cattle and geese^13^. However, various biotic and abiotic stresses, such as root-lesion nematodes^14^, fungal diseases^15–18^, viral infections^19^, ramie moth^20^, drought, flooding^21^, heavy metal contamination^22^, and nutrient deficiency^23^ often limit the productivity of ramie plants. At present, the detailed mechanisms underlying the ramie plants’ responses to these biotic and abiotic stresses are largely unknown. Using a variety of molecular tools, a few recent studies identified gene expression differences associated with ramie plants’ responses to drought, nutrient deficiency, cadmium (Cd) contamination, and infections by the fungal pathogen *Colletotrichum gloeosporioides*, the nematode *Pratylenchus coffeae*, and the ramie moth *Cocytodes coerulea* Guenée^12,24–33^. However, in their confirmations through qPCR, these studies used different reference genes and/or analyzed different plant tissues and/or stress conditions. As a result, it is difficult to compare observations from different studies.

The objectives of this study are to analyze the expression stability of a set of candidate reference genes in ramie plants under a variety of conditions and to identify the most suitable reference gene(s) for future qPCR analyses of gene expressions in this plant. Based on results from previous studies of ramie^24–27,30–33^ and in other plants^8–11,34^, the following eight genes showed relatively stable expressions under different conditions: *18S rRNA*, *ACT1*, *GAPDH*, *α-TUB*, *EF1α*, *UBQ*, *F-box*, and *CYP2*. We thus selected these genes for evaluation as potential reference genes for future studies of gene expression in ramie using qPCR. Specifically, the expression stability of these genes in the roots and leaves of ramie plants under different abiotic stresses (high temperature, low temperature, drought, Cd contamination, and high-salt stress), hormonal stimuli (salicylic acid [SA], Benzothiadiazole [BTH], methyl jasmonate [MeJA], ethephon [ETH], and gibberellin [GA3]), and biotic stress (infection by the parasitic oomycete *Pythium vexans*) were analyzed using four commonly used analytical programs (geNorm, NormFinder, BestKeeper, and RefFinder). Our analyses identified specific reference gene(s) for each given condition for future gene expression studies of ramie leaves and roots.

## Materials and Methods

### Plant sample preparation and treatment

An elite ramie cultivar, Zhongzhu No. 2, was used in this study. The seedlings were prepared via the stem cutting propagation method to obtain genetically identical seedlings of similar age and size^25^. The ramie seedlings were grown at 25 ± 1 °C with a 12 h photoperiod, 75 ± 1% relative humidity (RH), under light intensity of 5000 lux, in a greenhouse within the Institute of Bast Fiber Crops, Chinese Academy of Agricultural Science (IBFC, CAAS). When the seedlings were approximately 20 cm in height, they were used in the following treatments. For drought, salt, and heavy metal stress treatments, ramie plants were subjected to 200 mL of 20% PEG 8000, 200 mM of NaCl, and 400 µM CdCl_2_ for 4 hours, respectively. For cold and heat shock treatments, plants were transferred to a light incubator at temperatures of 4 °C and 40 °C for 4 h, respectively. For hormone treatments, ramie plants were subjected to MeJA (100 µM), SA (100 µM), BTH (100 µM), ETH (100 ppm), and GA_3_ (1 mM) respectively for 4 h, either by foliar spraying or by root soaking^8,34^. For the biotic stress treatment, ramie roots were inoculated with *P*. *vexans* isolate HF1 and incubated for three days using the method described previously^16^. All of the treatments were each performed in three biological replicates. Untreated plants grown for the same time intervals were collected as controls. The harvested plant materials (roots, leaves) from each of the treatments were washed with MINIQ-filtered water, immediately frozen in liquid nitrogen, and stored at −80 °C until use.

### Candidate reference genes selection and primer design

Eight candidate reference genes (*18S rRNA*, *ACT1*, *GAPDH*, *α-TUB*, *EF1α*, *UBQ*, *F-box*, and *CYP2*) were selected for this study based on two sets of criteria. The first was the relative stability of their expressions under different stresses, including drought, Cd, infection by root-lesion nematodes, and feeding by ramie moth larvae as revealed by transcriptome analyses using the next generation sequencing platform in ramie (Supplementary Table S1)^24–27,30–33^. The second criterion was that these eight genes had been used as internal reference genes for qPCR experiments in many other plants^8–11,34^. The sequences of the eight genes in ramie were obtained from either the NCBI database or the assembled transcriptome sequences described in previous studies^24–27,30,31,33^. Information about these eight genes is shown in Supplementary Table S2. Gene specific primers (Table 1) were designed using Primer3Plus (http://www.primer3plus.com/cgi-bin/dev/primer3plus.cgi)^35^.

**Table 1:** Information about the eight candidate reference genes and primer sequences used for quantitative real-time PCR (qPCR) in this study.

Gene symbol	Gene description	Gene ID	Primer sequences (5′-3′)^*^	Amplicon length (bp)	Tm (°C)	PCR efficiency (%)	Correlation coefficient (R^2^)
*18S*	18S rRNA	AF206870.1	tgacggagaattagggttcga/ccgtgtcaggattgggtaatt	100	60	99	0.9743
*ACT*	Actin1	DQ665832.2	gttgaaccctaaggctaacagag/ggaatccagcacgataccag	139	60	100	0.9944
*GAPDH*	glyceraldehyde-3- phosphate dehydrogenase	comp37700_c0	tggagacaagaaacagcaccct/cggcaattccgccatttaac	121	60	100	0.9635
*EF-1*α	Elongation factor 1α	comp36076_c0	tggccgtccttggaaatacc/ctcctgggcatcgtgacttt	121	60	99	0.9578
*αTUB*	α-tubulin	comp24081_c0	tgcatttcggtccacatcg/catcatcacctccgccaac	132	60	100	0.9222
*CYP2*	Cyclophilin2	comp26478_c0	cggcgagtctatctacgga/tgacgatttccattccctcg	200	60	100	0.9626
*UBQ*	Ubiquitin	comp20072_c0	agacgagcataacatttcctgc/gccgtactcttgccgattac	151	60	100	0.9273
*F-box*	F-box family protein	comp30228_c0	atagagggcgtaggctgagg/tccttcgggtggttatgttc	127	60	98	0.9889

^*^The primer sequences represent forward/reverse primers for each gene.

### RNA extraction and cDNA synthesis

For both root and leaf samples from each treatment, the total RNA was extracted using an EASYspin Plus Total RNA Kit (Aidlab, Beijing, China), following the manufacturer’s protocol. The concentration and purity of total RNA was determined using a NanoDrop 2000 spectrophotometer (Thermo Fisher Scientific, Waltham, MA, USA). RNA integrity was examined using an Agilent 2100 Bioanalyzer (Agilent, Santa Clara, CA, USA). First-strand cDNAs were synthesized from 1 μg of total RNA from the leaf or root samples, using the Transcriptor First Strand cDNA Synthesis Kit (Roche, Mannheim, Germany).

### qPCR amplification

To analyze expression stability of the eight candidate genes, we first determined the specificity and PCR efficiency for each of the designed primer pairs. To test the specificity of the primers to the target genes, PCR was performed using cDNA as the template. All PCR products were examined by agarose [1.5% (w/v)] gel electrophoresis. For each primer pair, the appearance of a single band of the expected size on the agarose gel was considered consistent with the primers being specific for the target gene. To test for PCR amplification efficiency, we followed the method described previously based on standard dilution curves in qPCR^36^, using the combined cDNA sample from the roots and leaves of control plants. All qPCR was performed using LightCycler 480 SYBR Green I Master, Roche LightCycler 96-well plates and Roche LightCycler 480 II (Roche Diagnostics, Mannheim, Germany). Each reaction mixture contained 10 μL SYBR Green I Master Mix, 3 μL diluted cDNA, 1 μL of forward primer (10 μM), 1 μL of reverse primer (10 μM), and 5 µL ddH_2_O in a total volume of 20 μL. The following amplification conditions were applied for all eight genes: 1 cycle at 95 °C for 10 min, 45 cycles at 95 °C for 10 s, 60 °C for 10 s, and 72 °C for 15 s, followed by 1 cycle of 95 °C for 10 s, 65 °C for 60 s and 95 °C for 1 s. RNase-free water was used as a negative control. In our analyses, each biological sample (i.e. a treatment x tissue combination) had three biological replicates, and each biological replicate had three technical replicates.

### Gene expression stability and statistical analysis

For each qPCR reaction, we obtained a Ct (threshold-cycle) value. The mean ± standard deviation (SD) of the Ct value for each tissue x treatment combination was calculated and the statistical significance of the Ct value differences between tissues and among treatments was obtained using the Student’s t-test. The Ct values for both types of tissues (i.e. leaves and roots) under the tested conditions were then analyzed by GeNorm^37^, NormFinder^38^, BestKeeper^39^ and RefFinder^40^ to evaluate and rank the expression stability of the eight candidate reference genes. These four programs were chosen because they use different formulae to calculate and rank expression stabilities and that they have been widely used to rank candidate reference genes for qPCR experiments across all major groups of organisms. Among the four programs, RefFinder is the most comprehensive. It is a web-based program that integrates four computational programs (geNorm, Normfinder, BestKeeper, and the comparative delta-Ct method) to compare and rank the tested candidate reference genes. Specifically, it uses the ranking information from each program, assigns an appropriate weight to each individual gene, and calculates the geometric mean of their weights for the overall final ranking^40^. Previous studies e.g.^34,36^ have found that these programs provide complementary information to help researchers in their final selection of reference genes for specific purposes.

## Results

### Specificity and amplification efficiency of qPCR primers

**Figure 1:**
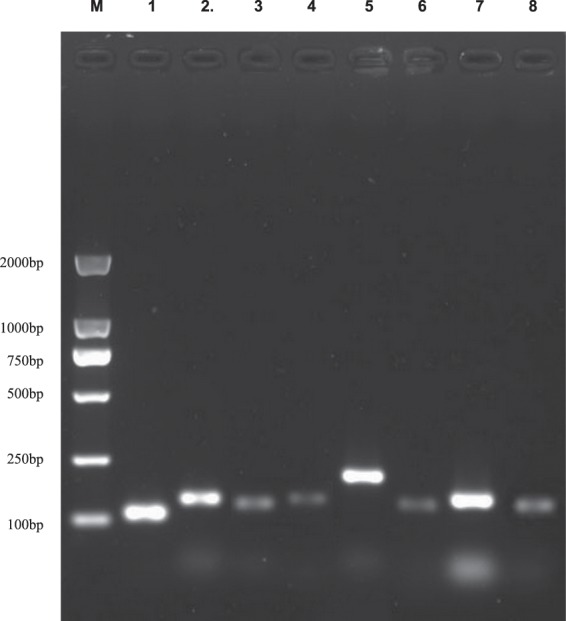
PCR products of eight candidate reference genes. Lane M: AL2000 DNA ladder, lane 1: *18S*, lane 2: ACT1, lane 3: GAPDH, lane 4: αTUB, lane 5: CYP, lane 6: F-box, lane 7: UBQ, lane 8: EF-1α.

The specificity of the gene-specific primers was determined by agarose gel electrophoresis of PCR amplicons and melting curve analysis. Agarose gel electrophoresis of PCR products of the eight candidate reference genes all showed a single band at the expected sizes (Fig. 1). In addition, each of the melting curves of these eight genes in ramie samples showed a single peak in qPCR, and the amplification curves of the nine repeats (three biological replicates x three technical replicates) for each gene had excellent repeatability (Supplementary Fig. S1). These results indicated that the primers were specific for their respective target genes. The PCR amplification efficiencies of the eight primer pairs ranged from 0.98 to 1.0 (Table 1), meeting the requirements for qPCR experiments. These results indicated that the primers for all eight candidate reference genes can be used for analyzing their expression patterns through qPCR.

### Expression profiling of candidate reference genes

**Figure 2:**
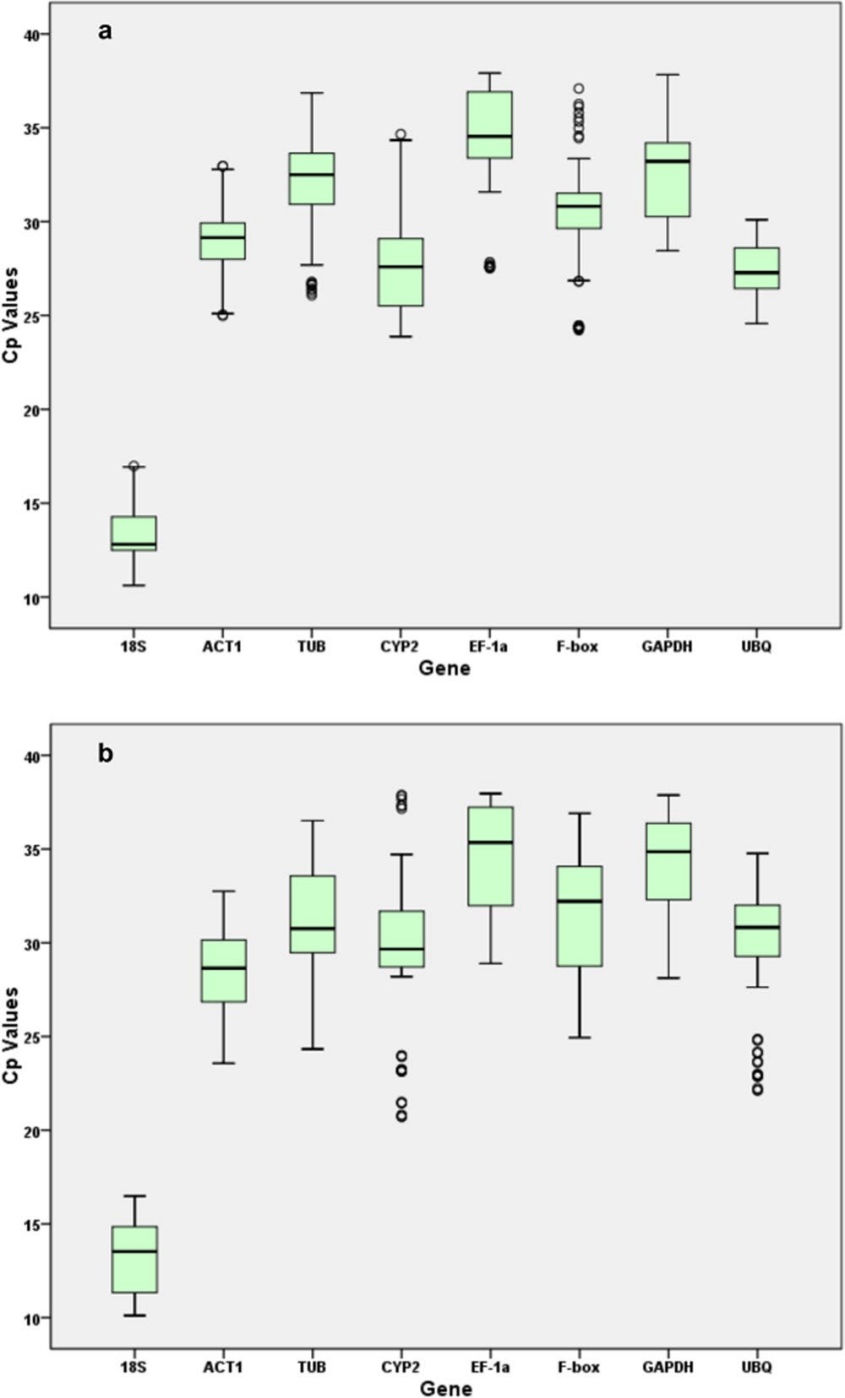
Box-plot graphs of Ct values for all candidate reference genes in leaves (**a**) and roots (**b**) of ramie under different conditions. Variations are displayed as medians (lines), 25^th^ to 75^th^ percent (boxes) and ranges (whiskers). The gene abbreviations are the same as in Table 1.

In qPCR, Ct values are used to quantify the expression levels of genes, with a low Ct value indicating a high gene expression level and vice versa. The expression profiles of the eight candidate reference genes in the roots and leaves of ramie in different treatments are summarized in Fig. 2. All eight candidate genes showed variations in expression levels, with different genes showing different ranges. Among the eight genes, the Ct values ranged from 9.6 to 37.9 among the root samples and 10.6 to 37.8 among the leaf samples. The *18S* rRNA gene showed the lowest average Ct values [13.44 ± 1.67 (mean ± standard deviation) in leaves and 13.36 ± 1.85 in roots], consistent with its high concentration in individual cells, while the *EF-1α* showed the highest average Ct value (34.47 ± 2.70 in the leaves and 34.66 ± 2.95 in the roots).

**Figure 3:**
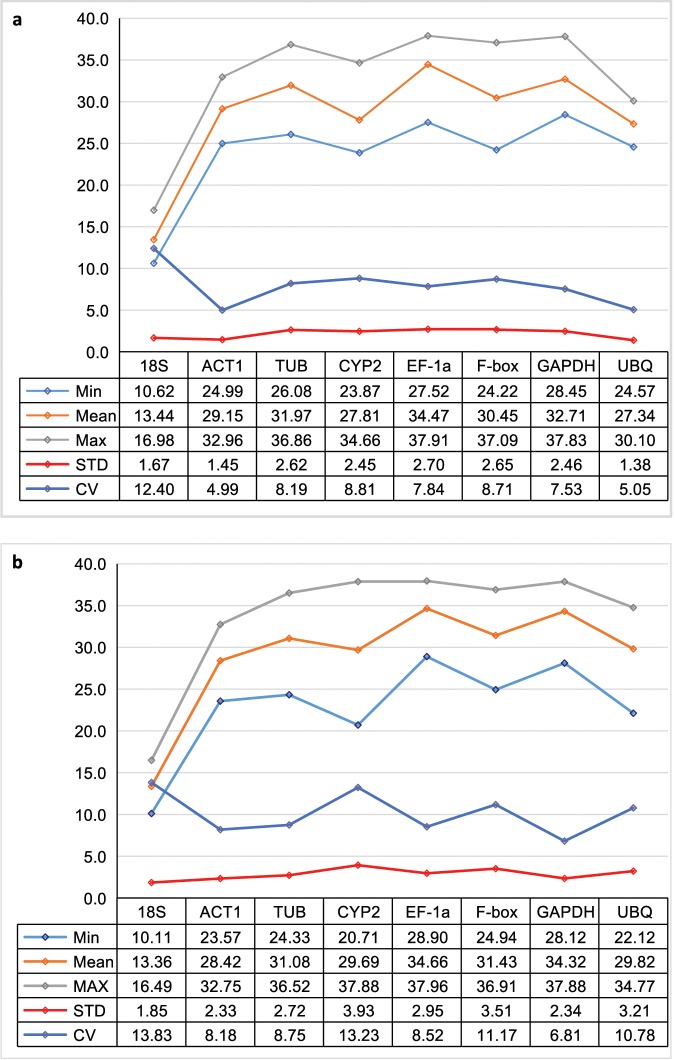
Data statistics of Ct values of eight candidate reference genes in leaves (**a**) and roots (**b**) of ramie. Total number of Ct values for each reference gene is 108.

Based on the means and standard deviations of Ct values, the overall coefficient of variation (CV) of the Ct values was calculated for each gene across all tested conditions. Low CV values indicated relatively low variability and high stability across the treatment conditions. The CV values of the eight candidate reference genes among all root samples were overall low and similar, ranging from 6.81% (for *GAPDH*) to 13.83% (for *18S rRNA*); among all leaf samples the range was from 4.99 (for *ACT1*) to 12.40% (for *18S rRNA*), respectively (Fig. 3). On the basis of CV values, the stability ranking of the eight candidate reference genes in root samples across all treatment conditions was: *GAPDH* > *ACT1* > *EF-1α* > *TUB* > *UBQ* > *F-box* > *CYP2* > *18S rRNA*, while in the leaf samples, the stability ranking was *ACT1* > *UBQ* > *GAPDH* > *EF-1α* > *TUB* > *F-box* > *CYP2* > *18S rRNA*. However, there were wide variations among treatments and groups of treatments in the relative rankings of expression stability among the eight genes. Below we summarize the main rankings by the four different analytical methods.

### Analysis of candidate reference genes by genorm

The stability of potential reference genes was first examined via geNorm software and the expression stability (M) of all eight candidates was calculated. A candidate gene with an M value <1.5 is considered to be a good reference gene for qPCR^37^. In all leaf samples, *ACT1* and *UBQ* were the most stably expressed genes, with the same M value of 1.13, while *EF-1α*, with an M value of 2.29, was the least stably expressed gene. Among the ramie samples subjected to abiotic stresses (including low and high temperatures, high salt, heavy metal, and drought), the *CYP2* and *F-box* genes were the most stably expressed in their leaves, both with an M value of 0.62, while the other six candidates showed less stable expression, all with M values over 1.73. In the leaf samples of ramie plants treated with different hormones (SA, BTH, MeJA and GA3), the *18S* rRNA and *ACT1* genes were the most stably expressed, both with an M value of 0.84. However, in the leaf samples of ramie infected with *P*. *vexans*, the *F-box* and *UBQ* genes were the most stably expressed, both having an M value of 0.54. For the leaves of ramie plants under other treatments, *18S rRNA*, *ACT1*, *UBQ*, and *CYP2* were the top ranked, showing relatively stable expression levels within individual treatments, while in most cases, the expression level of *GAPDH* was the least stable (Table 2).

**Table 2:** Suitability ranking, based on the geNorm program, of the eight candidate genes as potential reference genes for qPCR experiments involving leaves and roots of ramie plants under different treatments.

Tissue	Rank	Total	Abiotic	Hormone	*P*. *vexans*	SA	BTH	JA	ETH	GA3	Heat	Cold	NA	Cd	PEG
Leaf	1	ACT1 (1.13)	CYP2 (0.62)	18S (0.84)	F-box (0.54)	EF-1a (0.11)	18S (0.16)	ACT1 (0.15)	UBQ (0.18)	18S (0.42)	18S (0.10)	18S (0.21)	CYP2 (0.29)	CYP2 (0.40)	ACT1 (0.51)
	2	UBQ (1.13)	F-box (0.62)	ACT1 (0.84)	UBQ (0.54)	GAPDH (0.11)	ACT1 (0.16)	UBQ (0.15)	TUB (0.18)	F-box (0.42)	CYP2 (0.10)	TUB (0.21)	UBQ (0.29)	F-box (0.40)	GAPDH (0.51)
	3	GAPDH (1.71)	ACT1 (1.73)	UBQ (0.99)	EF-1a (0.71)	CYP2 (0.14)	F-box (0.18)	F-box (0.28)	CYP2 (0.21)	ACT1 (0.51)	UBQ (0.48)	EF-1a (0.32)	F-box (0.38)	EF-1a (0.50)	UBQ (0.64)
	4	TUB (1.91)	UBQ (1.83)	CYP2 (1.17)	18S (0.83)	TUB (0.16)	EF-1a (0.26)	18S (0.37)	F-box (0.26)	UBQ (0.52)	F-box (0.58)	CYP2 (0.41)	GAPDH (0.49)	ACT1 (0.63)	TUB (0.69)
	5	CYP2 (2.01)	GAPDH (2.04)	GAPDH (1.32)	GAPDH (0.89)	F-box (0.17)	TUB (0.32)	GAPDH (0.45)	GAPDH (0.38)	EF-1a (0.56)	ACT1 (0.70)	F-box (0.43)	EF-1a (0.56)	TUB (0.76)	EF-1a (0.77)
	6	F-Box (2.03)	TUB (2.2)	TUB (1.45)	TUB (0.95)	ACT1 (0.25)	CYP2 (0.45)	CYP2 (0.59)	18S (0.45)	TUB (0.60)	TUB (0.77)	GAPDH (0.47)	ACT1 (0.62)	UBQ (0.82)	18S (0.89)
	7	18S (2.19)	EF-1a (2.41)	F-box (1.69)	ACT1 (1.03)	18S (0.36)	UBQ (0.58)	TUB (0.75)	EF-1a (0.55)	CYP2 (0.68)	EF-1a (0.9)	ACT1 (0.57)	18S (0.73)	18S (0.96)	CYP2 (1.02)
	8	EF-1a (2.29)	18S (2.57)	EF-1a (1.79)	CYP2 (1.44)	UBQ (0.52)	GAPDH (0.71)	EF-1a (0.86)	ACT1 (0.76)	GAPDH (0.76)	GAPDH (1.07)	UBQ (0.63)	TUB (0.83)	GAPDH (1.26)	F-box (1.10)
Root	1	ACT1 (1.11)	F-box (1.08)	18S (1.18)	ACT1 (0.17)	TUB (0.19)	ACT1 (0.33)	EF-1a (0.3)	EF-1a (0.43)	GAPDH (0.37)	EF-1a (0.24)	F-box (0.14)	EF-1a (0.38)	ACT1 (0.35)	F-box (0.37)
	2	TUB (1.11)	UBQ (1.08)	UBQ (1.18)	TUB (0.17)	GAPDH (0.19)	CYP2 (0.33)	TUB (0.3)	CYP2 (0.43)	CYP2 (0.37)	TUB (0.24)	18S (0.14)	GAPDH (0.38)	CYP2 (0.35)	ACT1 (0.37)
	3	F-box (1.65)	ACT1 (1.54)	ACT1 (1.31)	F-box (0.24)	F-box (0.21)	TUB (0.39)	18S (0.35)	18S (0.51)	UBQ (0.42)	ACT1 (0.43)	ACT1 (0.23)	ACT1 (0.5)	EF-1a (0.43)	GAPDH (0.42)
	4	EF-1a (1.71)	EF-1a (1.57)	CYP2 (1.38)	EF-1a (0.39)	EF-1a (0.26)	18S (0.42)	UBQ (0.43)	GAPDH (0.58)	18S (0.58)	UBQ (0.49)	UBQ (0.38)	18S (0.68)	18S (0.54)	TUB (0.50)
	5	GAPDH (1.78)	TUB (1.69)	TUB (1.43)	CYP2 (0.57)	CYP2 (0.27)	UBQ (0.51)	GAPDH (0.47)	TUB (0.66)	F-box (0.83)	18S (0.56)	TUB (0.46)	TUB (0.85)	UBQ (0.66)	18S (0.71)
	6	18S (1.9)	GAPDH (1.79)	GAPDH (1.5)	UBQ (0.63)	ACT1 (0.33)	F-box (0.61)	F-box (0.58)	ACT1 (0.71)	EF-1a (0.94)	F-box (0.64)	CYP2 (0.5)	F-box (0.97)	GAPDH (0.79)	EF-1a (0.77)
	7	UBQ (1.98)	18S (1.91)	F-box (1.76)	GAPDH (0.72)	UBQ (0.41)	EF-1a (0.74)	ACT1 (0.67)	F-box (0.74)	ACT1 (1.03)	CYP2 (0.74)	EF-1a (0.54)	CYP2 (1.05)	F-box (0.95)	UBQ (0.80)
	8	CYP2 (2.08)	CYP2 (2.19)	EF-1a (1.9)	18S (0.81)	18S (0.49)	GAPDH (0.86)	CYP2 (0.89)	UBQ (0.79)	TUB (1.12)	GAPDH (1.01)	GAPDH (0.88)	UBQ (1.11)	TUB (1.14)	CYP2 (1.48)

For all samples of ramie roots, *ACT1* and *TUB* (both with an M value of 1.11) were the most stably expressed based on geNorm, while *CYP2* was the least stably expressed. In the root samples of ramie under abiotic stresses, *F-box* and *UBQ* were the most stably expressed genes, with the same M value of 1.08, while *CYP2* displayed the least stable expression with an M value of 2.19. For the ramie root samples under different hormonal treatments, the *18S rRNA* and *ACT1* were the most stably expressed genes, with the same M value of 0.84. In root samples infected with *P*. *vexans*, all eight candidate genes showed M values below 1.5; the ranking of the 8 genes based on M values was *ACT1*/*TUB* (0.17) < *F-box* (0.240) < *EF-1α* (0.39) < *CYP2* (0.57) < UBQ (0.63) < *GAPDH* (0.72) < *18S rRNA* (0.81). For the roots of ramie plants under other single treatments, *EF-1α* and *CYP2* were overall the most stably expressed, while in most cases *GAPDH* was the least stably expressed (Table 2).

**Figure 4:**
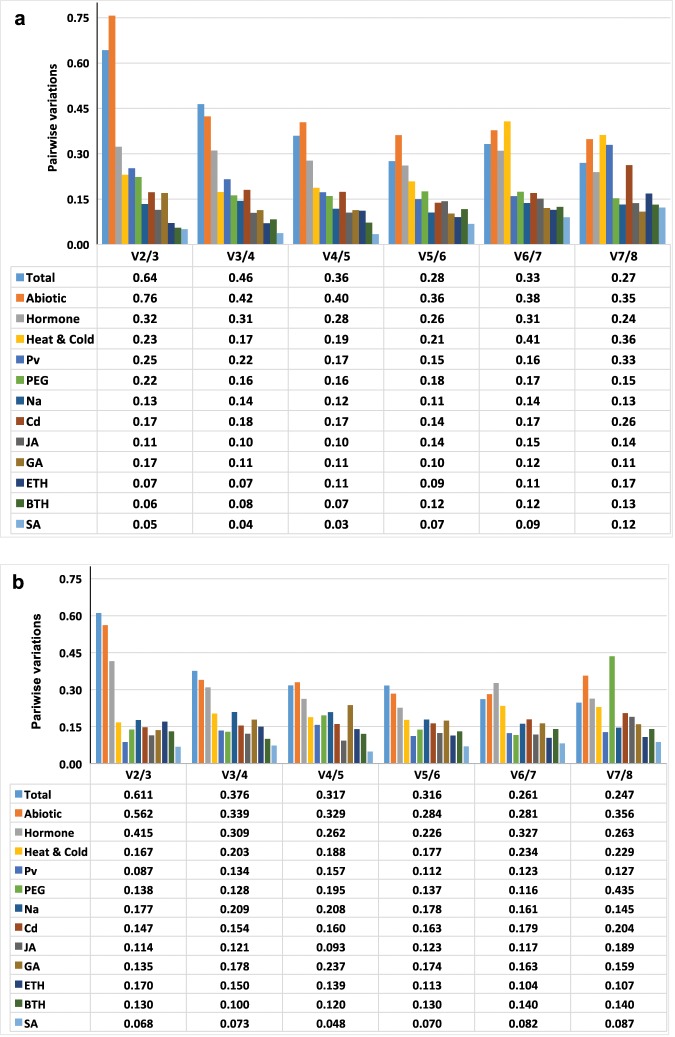
Determination of the optimal reference gene number by the geNorm program based on calculations using the pairwise variation (Vn/Vn + 1) approach. The optimal number of reference genes (n) is reached when Vn/Vn + 1 < 0.15. (**a**) leaves; (**b**) roots.

To determine the optimal number of reference genes for gene expression studies, we performed a stepwise calculation of the pairwise variation (V_n_/V_n_ + 1) between sequential normalization factors using geNorm. In this analysis, a V_n_/V_n_ + 1 < 0.15 indicates that introducing an additional reference gene for normalization is not necessary^37^. Among the eight genes, the most stable reference gene sets varied among samples and treatments (Fig. 4). For the leaf samples of ramie plants subjected to SA, BTH, ETH, GA, and cold or high salt conditions, the V_2_/V_3_ values were all lower than 0.15, indicating that two reference genes were sufficient for normalization. For leaf samples treated with PEG, infected with *P*. *vexans*, under abiotic stress, or subjected to a hormonal treatment, no V_n_/V_n+1_ value was <0.15, i.e. no optimal reference gene number was suggested by the program geNorm (Fig. 4a). A variable pattern was also noted for the root samples. For example, in the root samples of ramie plants subjected to SA, BTH, GA3, or JA treatments or under Cd, PEG, or cold stresses, the V_2_/V_3_ values were all lower than 0.15, indicating that two reference genes should be used for normalization. In contrast, for the root samples of ramie plants subjected to high temperature stress or stimulated by ETH, three and four reference genes were recommended for qPCR analyses of gene expressions, respectively (Fig. 4b).

### Analysis of reference genes by NormFinder

Analyses using the model-based approach NormFinder revealed the stability value of each gene under various treatments. Overall, the most stable reference gene for all the leaf and root samples was *ACT1*, with stability values of 0.658 and 0.382, respectively. Individually, *ACT1* was the most stably expressed gene in both the leaf and root samples under abiotic stresses. However, under hormonal treatments, *CYP2* (0.419) and *TUB* (0.407) were the most stably expressed genes for leaves and roots, respectively. In the leaf and root samples of ramie plants infected by *P*. *vexans* or subjected to cold or heat stress, *F-box* and *ACT1* were the most stably expressed genes, respectively. Based on NormFinder, *GAPDH* showed among the least stable expression under most conditions, both in the root and leaf samples (Table 3). Among individual treatments, the most suitable reference genes identified by NormFinder were similar to those selected by geNorm except for the GA3-treated plants for both the leaf and root samples. Thus, the reference genes recommended by NormFinder were highly consistent with those obtained by geNorm analysis.

**Table 3:** Suitability ranking, based on the NormFinder program, of the eight candidate genes as potential reference genes for qPCR experiments.

Tissue	Rank	Total	Abiotic	Hormone	*P*.*vexans*	SA	BTH	JA	ETH	GA3	Heat	Cold	NA	Cd	PEG
Leaf	1	ACT1 (0.66)	ACT1 (0.52)	CYP2 (0.42)	F-box (0.13)	GAPDH (0.04)	ACT1 (0.05)	18S (0.13)	UBQ (0.05)	ACT1 (0.12)	18S (0.04)	CYP2 (0.07)	CYP2 (0.07)	TUB (0.24)	EF-1a (0.18)
	2	UBQ (0.93)	UBQ (0.59)	GAPDH (0.43)	UBQ (0.19)	CYP2 (0.05)	F-box (0.10)	ACT1 (0.31)	TUB (0.06)	UBQ (0.18)	CYP2 (0.04)	F-box (0.17)	UBQ (0.10)	F-box (0.42)	TUB (0.24)
	3	GAPDH (1.04)	F-box (1.15)	TUB (0.55)	EF-1a (0.39)	ACT1 (0.11)	18S (0.17)	UBQ (0.35)	CYP2 (0.21)	EF-1a (0.24)	UBQ (0.37)	18S (0.22)	F-box (0.31)	ACT1 (0.45)	UBQ (0.40)
	4	F-box (1.12)	GAPDH (1.18)	ACT1 (0.78)	18S (0.46)	EF-1a (0.13)	EF-1a (0.19)	GAPDH (0.37)	GAPDH (0.25)	TUB (0.31)	F-box (0.51)	TUB (0.25)	EF-1a (0.44)	CYP2 (0.45)	GAPDH (0.53)
	5	CYP2 (1.15)	CYP2 (1.39)	18S (1.10)	TUB (0.54)	TUB (0.18)	TUB (0.22)	CYP2 (0.39)	F-box (0.27)	F-box (0.37)	TUB (0.56)	EF-1a (0.30)	ACT1 (0.45)	UBQ (0.55)	ACT1 (0.60)
	6	TUB (1.19)	EF-1a (1.49)	UBQ (1.17)	GAPDH (0.63)	F-box (0.24)	CYP2 (0.42)	F-box (0.43)	18S (0.27)	18S (0.44)	EF-1a (0.61)	ACT1 (0.46)	GAPDH (0.49)	18S (0.59)	CYP2 (0.71)
	7	EF-1a (1.43)	TUB (1.76)	F-box (1.27)	ACT1 (0.94)	18S (0.44)	UBQ (0.63)	TUB (0.68)	EF-1a (0.64)	GAPDH (0.59)	ACT1 (0.74)	GAPDH (0.46)	18S (0.53)	EF-1a (0.66)	18S (0.74)
	8	18S (1.46)	18S (1.87)	EF-1a (1.28)	CYP2 (1.82)	UBQ (0.67)	GAPDH (0.72)	EF-1a (0.74)	ACT1 (0.93)	CYP2 (0.61)	GAPDH (1.05)	UBQ (0.52)	TUB (0.72)	GAPDH (1.43)	F-box (0.76)
Root	1	ACT1 (0.38)	ACT1 (0.22)	TUB (0.41)	ACT1 (0.07)	TUB (0.13)	CYP2 (0.03)	18S (0.11)	EF-1a (0.17)	18S (0.18)	TUB (0.08)	EF-1a (0.02)	ACT1 (0.24)	ACT1 (0.12)	ACT1 (0.13)
	2	TUB (0.75)	EF-1a (0.65)	CYP2 (0.52)	TUB (0.11)	GAPDH (0.13)	18S (0.13)	EF-1a (0.13)	CYP2 (0.21)	GAPDH (0.43)	ACT1 (0.14)	F-box (0.03)	F-box (0.46)	CYP2 (0.2)	F-box (0.13)
	3	GAPDH (0.94)	F-box (0.88)	ACT1 (0.55)	F-box (0.13)	F-box (0.15)	ACT1 (0.27)	TUB (0.27)	18S (0.33)	F-box (0.48)	EF-1a (0.15)	ACT1 (0.07)	EF-1a (0.54)	EF-1a (0.26)	GAPDH (0.15)
	4	UBQ (1.08)	UBQ (0.93)	GAPDH (0.66)	EF-1a (0.39)	ACT1 (0.17)	F-box (0.34)	UBQ (0.34)	GAPDH (0.40)	EF-1a (0.51)	UBQ (0.20)	UBQ (0.18)	GAPDH (0.59)	UBQ (0.41)	TUB (0.18)
	5	F-box (1.09)	TUB (0.99)	UBQ (1.18)	CYP2 (0.45)	CYP2 (0.24)	TUB (0.40)	GAPDH (0.35)	TUB (0.41)	ACT1 (0.63)	18S (0.36)	18S (0.23)	TUB (0.59)	18S (0.52)	18S (0.68)
	6	EF-1a (1.14)	GAPDH (1.09)	18S (1.20)	UBQ (0.51)	EF-1a (0.27)	UBQ (0.55)	ACT1 (0.50)	ACT1 (0.42)	CYP2 (0.71)	F-box (0.53)	TUB (0.47)	CYP2 (0.62)	GAPDH (0.57)	EF-1a (0.71)
	7	CYP2 (1.32)	18S (1.52)	F-box (1.33)	GAPDH (0.59)	UBQ (0.33)	EF-1a (0.63)	F-box (0.53)	F-box (0.55)	UBQ (0.73)	CYP2 (0.79)	CYP2 (0.53)	18S (0.65)	F-box (0.95)	UBQ (0.79)
	8	18S (1.32)	CYP2 (1.94)	EF-1a (1.42)	18S (0.69)	18S (0.48)	GAPDH (0.77)	CYP2 (1.04)	UBQ (0.58)	TUB (0.86)	GAPDH (1.25)	GAPDH (1.31)	UBQ (0.78)	TUB (1.12)	CYP2 (2.41)

### Analysis of reference genes by BestKeeper

The excel-based BestKeeper algorithm was also used to evaluate the expression stability of the eight candidate reference genes. This approach initially uses data on the standard deviation (SD) and co-efficient of variation (CV) of the average Ct values for the specific treatments. The lower the SD and CV values, the more stable its expression is among the treatments. In this analysis, genes with SD > 1 were considered as undesirable reference genes. The genes with SD values less than 1 were then analyzed to derive a BestKeeper Index. When all the treatments were considered together, the Ct SD values were all greater than 1 for both the leaf and root samples. Thus, our results based on this criterium alone suggested that no single gene could be used as the reference gene across all the treatments. However, there were big variations among the genes for subsets of the samples and tested conditions, with some showing greater promise as candidate reference genes than others. For example, in ramie leaves stimulated by hormonal treatments and under abiotic stresses, both *18S rRNA* and *UBQ* showed low SD values and relatively stable expression levels. For root samples, none of the eight genes showed stable expression in groups of ramie plants subjected to hormonal treatments, under abiotic stresses, or in the total samples. However, among the single treatments, genes *18S rRNA*, *EF-1α*, *F-box*, and TUB showed relatively stable expression (SD < 1) in most cases. In contrast, genes *CYP2*, *UBQ*, and *GAPDH* displayed relatively unstable expression under many conditions, for both the leaf and root samples (Table 4). Taken together, results from BestKeeper analyses suggest that different treatments will require separate considerations for reference genes in qPCR experiments (Table 4).

**Table 4:** Ranking the expression stabilities of eight candidate reference genes in ramie leaves and roots based on the Bestkeeper program.

Tissue	Rank	Total	Abiotic	Hormone	*P*. *vexans*	SA	BTH	JA	ETH	GA3	Heat	Cold	NA	Cd	PEG
Leaf	1	ACT1 1.09 ± 3.75	UBQ 0.92 ± 3.34	18S18S 0.44 ± 3.55	F-box 0.36 ± 1.12	TUB 0.02 ± 0.08	F-box 0.19 ± 0.63	ACT1 0.10 ± 0.35	CYP2 0.37 ± 1.47	18S 0.33 ± 2.55	CYP2 0.25 ± 0.94	ACT1 0.12 ± 0.44	18S 0.15 ± 1.15	CYP2 0.31 ± 1.10	EF-1a 0.12 ± 0.31
	2	UBQ 1.12 ± 4.10	ACT1 1.16 ± 3.89	ACT1 0.82 ± 2.87	UBQ 0.48 ± 1.64	F-box 0.05 ± 0.21	EF-1a 0.22 ± 0.64	GAPDH 0.12 ± 0.37	UBQ 0.37 ± 1.49	F-box 0.39 ± 1.24	TUB 0.37 ± 1.08	TUB 0.19 ± 0.62	TUB 0.31 ± 0.95	Box 0.34 ± 1.09	TUB 0.39 ± 1.10
	3	18S 1.23 ± 9.22	18S 1.30 ± 8.91	UBQ 1.08 ± 4.00	18S 0.50 ± 3.77	EF-1a 0.09 ± 0.31	18S 0.24 ± 1.91	UBQ 0.16 ± 0.57	ACT1 0.38 ± 1.40	UBQ 0.43 ± 1.61	EF-1a 0.56 ± 1.52	EF-1a 0.24 ± 0.70	UBQ 0.42 ± 1.53	GAPDH 0.41 ± 1.28	F-box 0.62 ± 1.73
	4	CYP2 1.76 ± 6.32	EF-1a 1.52 ± 4.20	CYP2 1.34 ± 5.02	TUB 0.52 ± 1.58	GAPDH 0.15 ± 0.49	ACT1 0.25 ± 0.84	F-box 0.19 ± 0.62	TUB 0.41 ± 1.34	CYP2 0.58 ± 2.10	UBQ 0.74 ± 2.69	CYP2 0.36 ± 1.27	CYP2 0.66 ± 2.24	ACT1 0.48 ± 1.61	CYP2 0.71 ± 2.13
	5	F-box 1.83 ± 6.00	F-box 2.11 ± 6.79	TUB 1.54 ± 4.95	GAPDH 0.57 ± 1.71	CYP2 0.15 ± 0.59	TUB 0.30 ± 0.91	18S 0.21 ± 1.69	GAPDH 0.49 ± 1.58	GAPDH 0.60 ± 1.78	18S 0.78 ± 4.76	18S 0.50 ± 4.34	F-box 0.83 ± 2.67	EF-1a 0.6 ± 1.76	18S 0.72 ± 5.13
	6	GAPDH 1.93 ± 5.93	GAPDH 2.25 ± 6.76	GAPDH 1.55 ± 4.77	EF-1a 0.67 ± 1.85	ACT1 0.37 ± 1.32	GAPDH 0.58 ± 1.72	CYP2 0.74 ± 2.64	F-box 0.51 ± 1.69	ACT1 0.70 ± 2.42	F-box 0.80 ± 2.70	GAPDH 0.55 ± 1.89	EF-1a 0.87 ± 2.52	18S 0.67 ± 4.83	UBQ 0.81 ± 2.84
	7	EF-1a 2.01 ± 5.83	CYP2 2.30 ± 8.08	F-box 2.08 ± 7.05	ACT1 0.77 ± 2.52	18S 0.65 ± 4.96	CYP2 0.80 ± 2.86	TUB 0.89 ± 2.74	EF-1a 0.58 ± 1.76	EF-1a 0.83 ± 2.45	ACT1 0.92 ± 3.08	UBQ 0.60 ± 2.33	GAPDH 0.97 ± 2.96	TUB 0.94 ± 2.67	GAPDH 0.85 ± 2.34
	8	TUB 2.01 ± 6.30	TUB 2.53 ± 7.74	EF-1a 2.12 ± 6.49	CYP2 0.94 ± 3.26	UBQ 0.89 ± 3.23	UBQ 1.04 ± 3.79	EF-1a 0.94 ± 2.64	18S 0.66 ± 5.68	TUB 0.86 ± 2.65	GAPDH 0.94 ± 2.65	F-box 0.76 ± 2.53	ACT1 1.03 ± 3.62	UBQ 1.14 ± 4.11	ACT1 0.93 ± 2.95
Root	1	18S 1.60 ± 11.96	18S 1.62 ± 12.28	UBQ 1.22 ± 3.96	F-box 0.30 ± 0.93	TUB 0.04 ± 0.14	F-box 0.20 ± 0.63	TUB 0.23 ± 0.67	EF-1a 0.01 ± 0.03	18S 0.20 ± 1.32	EF-1a 0.22 ± 0.73	18S 0.03 ± 0.28	EF-1a 0.13 ± 0.35	18S 0.11 ± 0.86	CYP2 0.08 ± 0.21
	2	ACT1 1.88 ± 6.64	GAPDH 2.48 ± 7.30	CYP2 1.36 ± 4.45	EF-1a 0.31 ± 0.86	F-box 0.05 ± 0.20	GAPDH 0.30 ± 0.82	EF-1a 0.26 ± 0.71	CYP2 0.24 ± 0.81	F-box 0.34 ± 1.00	TUB 0.26 ± 0.89	F-box 0.09 ± 0.35	ACT1 0.25 ± 0.80	F-box 0.22 ± 0.65	EF-1a 0.13 ± 0.34
	3	GAPDH 1.96 ± 5.71	ACT1 2.82 ± 9.97	18S 1.41 ± 10.07	TUB 0.33 ± 1.10	GAPDH 0.09 ± 0.27	18S 0.32 ± 2.22	UBQ 0.28 ± 0.88	18S 0.32 ± 3.03	EF-1a 0.42 ± 1.12	18S 0.51 ± 4.45	ACT1 0.25 ± 1.03	GAPDH 0.40 ± 1.09	EF-1a 0.35 ± 1.00	UBQ 0.20 ± 0.61
	4	TUB 2.21 ± 7.10	EF-1a 3.12 ± 9.14	ACT1 1.53 ± 5.34	ACT1 0.38 ± 1.34	CYP2 0.18 ± 0.62	EF-1a 0.38 ± 1.09	18S 0.45 ± 3.01	GAPDH 0.33 ± 0.97	GAPDH 0.50 ± 1.35	ACT1 0.51 ± 2.00	EF-1a 0.43 ± 1.41	18S 0.60 ± 3.74	ACT1 0.53 ± 1.80	18S 0.27 ± 1.89
	5	EF-1a 2.39 ± 6.91	TUB 3.20 ± 10.32	GAPDH 1.54 ± 4.40	UBQ 0.44 ± 1.46	EF-1a 0.19 ± 0.64	CYP2 0.51 ± 1.69	GAPDH 0.50 ± 1.41	ACT1 0.50 ± 1.91	CYP2 0.73 ± 2.31	UBQ 0.68 ± 2.82	UBQ 0.50 ± 2.16	TUB 0.83 ± 2.37	CYP2 0.72 ± 2.25	TUB 0.66 ± 2.01
	6	UBQ 2.43 ± 8.14	F-box 4.17 ± 13.39	TUB 1.65 ± 5.23	GAPDH 0.47 ± 1.34	ACT1 0.35 ± 1.27	TUB 0.65 ± 2.09	ACT1 0.59 ± 1.91	TUB 0.53 ± 1.76	ACT1 0.74 ± 2.51	F-box 0.96 ± 3.57	TUB 0.64 ± 2.51	F-box 0.90 ± 2.62	GAPDH 0.99 ± 2.76	ACT1 0.78 ± 2.55
	7	F-box 2.81 ± 8.95	UBQ 4.29 ± 14.90	EF-1a 2.20 ± 6.34	CYP2 0.61 ± 2.04	UBQ 0.38 ± 1.22	ACT1 0.70 ± 2.41	F-box 0.69 ± 1.98	UBQ 0.57 ± 2.00	UBQ 0.76 ± 2.45	GAPDH 1.04 ± 3.32	CYP2 0.75 ± 3.33	CYP2 1.16 ± 3.52	UBQ 1.03 ± 3.20	GAPDH 0.89 ± 2.48
	8	CYP2 3.00 ± 10.02	CYP2 5.58 ± 18.83	F-box 2.36 ± 7.51	18S 0.96 ± 7.64	18S 0.61 ± 4.24	UBQ 0.97 ± 3.13	CYP2 1.58 ± 4.90	F-box 0.62 ± 2.00	TUB 0.88 ± 2.64	CYP2 1.17 ± 5.16	GAPDH 1.58 ± 5.16	UBQ 1.26 ± 3.91	TUB 1.84 ± 5.65	F-box 0.95 ± 2.63

Note: Data after gene symbols are Std ± CV%.

### Analysis of reference genes by RefFinder

As described briefly above, RefFinder is a comprehensive web-based tool for reference gene identification that integrates geNorm, NormFinder, Delta Ct and BestKeeper approaches^40^. In this study, RefFinder was employed to evaluate the eight candidate reference genes in leaf and root samples under different conditions. The result showed that *ACT1* was the most stably expressed gene when all the leaf and root samples were considered together. Individually, it was also the most stable one in the ramie leaf and root samples under various abiotic stresses. However, under other conditions, different reference genes were recommended by RefFinder. For example, in ramie treated with different hormones, *CYP2* and *TUB* showed the most stable expression in leaf and root samples, respectively. In the leaves and roots of ramie plants infected with *P*. *vexans*, *UBQ* and *TUB* were the top ranked reference genes respectively. In most other single treatments, *ACT1*, *CYP2*, and *UBQ* displayed relatively stable expressions in leaves, while *EF-1α*, *TUB*, and *ACT1* showed relatively stable expression in roots. *GAPDH* displayed the least stable expression in most cases for both the leaf and root samples (Table 5).

**Table 5:** Ranking the expression stabilities of eight candidate reference genes in ramie leaves and roots based on the RefFinder program.

Tissue	Rank	Total	Abiotic	Hormone	*P*.*vexans*	SA	BTH	JA	ETH	GA3	Heat	Cold	NA	Cd	PEG
Leaf	1	ACT1 (1.00)	ACT1 (1.57)	CYP2 (3.50)	UBQ (1.19)	GAPDH (1.5)	ACT1 (1.41)	ACT1 (1.41)	UBQ (1.19)	ACT1 (1.97)	18S (1.19)	TUB (1.86)	CYP2 (1.41)	box (1.41)	EF-1a (1.50)
	2	UBQ (1.68)	UBQ (2.00)	18S (6.73)	F-box (1.41)	EF-1a (2.45)	box (1.86)	18S (1.86)	TUB (1.57)	UBQ (2.38)	CYP2 (1.41)	CYP2 (2.11)	UBQ (1.86)	CYP2 (1.68)	TUB (2.38)
	3	GAPDH (3.83)	F-box (2.59)	ACT1 (1.57)	18S (3.46)	CYP2 (2.63)	18S (2.06)	UBQ (2.06)	CYP2 (2.71)	18S (2.45)	UBQ (3.41)	18S (2.63)	F-box (3.41)	TUB (2.94)	GAPDH (3.25)
	4	F-box (4.43)	CYP2 (3.50)	GAPDH (4.86)	EF-1a (3.83)	TUB (2.99)	EF-1a (3.72)	F-box (4.36)	F-box (4.23)	F-box (2.94)	F-box (4.43)	F-box (3.31)	18S (4.30)	ACT1 (3.46)	UBQ (3.57)
	5	CYP2 (5.00)	GAPDH (4.86)	TUB (6.96)	TUB (4.95)	F-box (4.16)	TUB (5.00)	GAPDH (4.47)	GAPDH (4.73)	EF-1a (4.05)	TUB (4.95)	EF-1a (3.87)	EF-1a (4.86)	EF-1a (4.53)	ACT1 (3.76)
	6	TUB (5.63)	EF-1a (5.24)	UBQ (2.00)	GAPDH (5.48)	ACT1 (5.05)	CYP2 (6.24)	CYP2 (5.73)	18S (6.24)	TUB (5.09)	EF-1a (5.24)	GAPDH (4.74)	GAPDH (5.42)	UBQ (5.73)	CYP2 (5.42)
	7	18S (5.86)	18S (6.73)	F-box (2.59)	ACT1 (7.00)	18S (7.00)	UBQ (7.24)	TUB (7.00)	EF-1a (6.74)	GAPDH (6.51)	ACT1 (6.44)	ACT1 (6.74)	TUB (5.66)	18S (6.74)	F-box (5.86)
	8	EF-1a (7.20)	TUB (6.96)	EF-1a (2.54)	CYP2 (8.00)	UBQ (8.00)	GAPDH (7.44)	EF-1a (8.00)	ACT1 (8.00)	CYP2 (7.48)	GAPDH (8.00)	UBQ (8.00)	ACT1 (5.89)	GAPDH (8.00)	18S (6.88)
Root	1	ACT1 (1.19)	ACT1 (1.73)	TUB (2.34)	TUB (1.41)	TUB (1.57)	CYP2 (1.41)	EF-1a (1.19)	EF-1a (1.19)	18S (1.41)	TUB (1.19)	F-box (1.68)	EF-1a (1.57)	ACT1 (1.32)	ACT1 (1.78)
	2	TUB (2.00)	F-box (2.71)	UBQ (2.45)	ACT1 (1.57)	F-box (1.73)	18S (2.38)	TUB (2.06)	CYP2 (1.41)	GAPDH (2.00)	EF-1a (1.86)	18S (2.11)	ACT1 (1.57)	CYP2 (2.00)	F-box (1.93)
	3	GAPDH (3.41)	EF-1a (2.83)	18S (2.66)	F-box (2.28)	GAPDH (1.86)	ACT1 (2.71)	18S (2.21)	18S (3.00)	F-box (3.08)	ACT1 (2.63)	ACT1 (2.28)	GAPDH (2.63)	EF-1a (2.71)	TUB (3.72)
	4	18S (4.14)	UBQ (3.25)	CYP2 (2.91)	EF-1a (4.23)	CYP2 (4.73)	F-box (3.31)	UBQ (3.72)	GAPDH (4.23)	CYP2 (3.83)	UBQ (3.94)	EF-1a (3.44)	F-box (3.83)	18S (3.16)	EF-1a (3.83)
	5	F-box (4.68)	18S (4.30)	ACT1 (3.08)	CYP2 (5.23)	EF-1a (4.90)	TUB (4.16)	GAPDH (5.00)	ACT1 (5.18)	EF-1a (4.12)	18S (4.40)	UBQ (3.94)	TUB (5.23)	UBQ (4.68)	GAPDH (3.83)
	6	UBQ (5.14)	GAPDH (4.56)	GAPDH (4.43)	UBQ (5.42)	ACT1 (5.18)	EF-1a (5.66)	F-box (6.24)	TUB (5.48)	ACT1 (5.44)	F-box (6.00)	TUB (5.73)	18S (5.29)	GAPDH (6.24)	18S (3.98)
	7	EF-1a (5.42)	TUB (5.00)	F-box (7.24)	GAPDH (7.00)	UBQ (7.00)	UBQ (6.16)	ACT1 (6.74)	F-box (7.24)	UBQ (5.66)	CYP2 (7.24)	CYP2 (6.74)	CYP2 (6.19)	F-box (6.44)	UBQ (5.66)
	8	CYP2 (7.74)	CYP2 (8.00)	EF-1a (7.74)	18S (8.00)	18S (8.00)	GAPDH (7.74)	CYP2 (8.00)	UBQ (7.74)	TUB (8.00)	GAPDH (7.74)	GAPDH (8.00)	UBQ (8.00)	TUB (8.00)	CYP2 (8.00)

Using RefFinder, we also analyzed and ranked the suitable reference genes across root and leaf samples. As shown in Supplementary Table S3, *ACT1*, *F-box*, and *18S* rRNA were ranked as the most suitable reference genes under most conditions (hormone stimuli, hot, cold, high salt, or heavy metal stress, and *P*. *vexans* infection), while *CYP2* and *UBQ* were ranked the least suitable for most of the treatments.

## Discussion

Ramie is an excellent source of long, natural fiber. It has attracted increasing attention from farmers, consumers and researchers in many parts of the globe. In China, many types of research are being conducted on ramie, including germplasm collection and assessment, domestication, and breeding for high yield, long fiber, and stress tolerance^24,25,41–44^. One of the emerging topics of research is to identify the key genes and metabolic pathways involved in ramie’s growth and its response to environmental stresses^45–50^. Gene expression profiling represents an important approach for understanding the roles of various genes during these processes. qPCR is a reliable and sensitive technique for measuring gene expression levels. However, accurate interpretations of qPCR results depend on the stability of reference genes used for data normalization. So far, several genes have been used as reference genes for normalizing gene expression data in ramie for a few selected conditions. However, the appropriateness of these genes as references has not been critically evaluated^30–32^. Indeed, previous studies in other organisms suggested that different tissues, different developmental stages and different environmental conditions may require different reference genes in order to accurately interpret the expressions of specific genes in qPCR experiments^8–11^. In this study, we screened eight candidate genes in ramie for their potential use as reference genes. These eight genes were chosen based on the ramie transcriptome data that showed limited variations in their relative abundance when ramie plants were subjected to several selected treatments, such as drought, Cd, ramie moth and root lesion nematode infestations. In addition, all eight genes have been used as reference genes for qPCR analyses of gene expression data in other plants, with several (e.g. *ACT1*, *TUB*) being commonly used across the Eukaryotic Domain.

Generally, an ideal reference gene is one that is stably expressed in different tissues of different cultivars under a wide range of environmental conditions. Based on the combined rankings of four programs, among the eight genes, on an individual gene basis, *ACT1* showed overall the most stable expression in leaf and root samples. Thus, if only a single gene were to be used as an internal reference, *ACT1* would be the most suitable reference gene for both leaf and root samples under a variety of conditions. Indeed, actin family genes are well-known reference genes across the Eukaryote Domain. However, our analyses indicated that for certain conditions/tissues, another gene or a combination of genes was more suitable as an internal reference in order to provide accurate normalization in qPCR experiments. For example, in ramie plants stimulated by hormones, *TUB* showed the highest stability and should be selected as the reference gene for analyzing gene expressions in the roots. Similar findings were reported for other plants, with genes displaying variable stability under different conditions. For example, when tomato leaves were subjected to treatments of nitrogen (N) starvation, low temperature, and suboptimal light during growth, among their eight screened candidate genes [*ACT1*, *TUB*, *EF1*, *GAPDH*, phosphoglycerate kinase (*PGK*), ribosomal protein L2 (*RPL2*), ubiquitin (*UBI*), and a catalytic subunit of protein phosphatase 2 A (*PP2Acs*)], *GAPDH* and *PGK* ranked at the top during light stress but poorly during N starvation and cold stress^51^. In contrast, *EF1* ranked the best during N starvation and cold stress but poorly during light stress. Four genes *ACT1*, *UBI*, *RPL2*, and *PP2Acs* all appeared to be relatively stably expressed when all stress conditions were considered. Similar to what we found, no gene in tomato was identified that exhibited such a constant level of expression as to outperform all other candidates under all individual experimental conditions^51^. Indeed, similar results were also found for carrot leaves and roots^8,52^. In one study, *ACT1* and *TUB* were determined to be the most suitable reference genes for carrot leaves under different abiotic stresses and hormone stimuli^8^. In another, among the nine screened candidates, *ACT1* showed overall the most stable expression in carrot roots and leaves while *GAPDH* was the least stable^52^. Their overall analyses suggested *ACT1* or different combinations of *ACT1*, *EF-1α*, the eukaryotic translation initiation factor 4α (*eIF-4α*), *TUB*, or *UBQ* were needed to normalize gene expression during carrot development^52^.

For both the leaf and root samples, there was no unanimity among the four analytical programs in ranking the most suitable reference gene(s) across all treatments. However, in most cases, one candidate gene was usually ranked as the most suitable reference gene by two to three algorithms, indicating that they were potentially good reference genes for these treatments. The degree of agreement for specific sets of treatments ranged from unanimity to complete disagreement. For instance, in the roots of ramie treated by SA or ETH, *TUB* and *EF-1a* were determined to be the most suitable reference genes, respectively, by all four analytical methods, indicating that these should be the preferred reference genes in these environmental conditions. In contrast, in the leaves of ramie under cold stress, *18S rRNA*, *CYP2*, *ACT1*, and *TUB* were ranked as the most suitable reference genes according to geNorm, NormFinder, BestKeeper, and RefFinder, respectively.

While environmental influences on gene expression are well documented, even for reference genes, in recent years, emerging evidence indicates that most genes also show tissue specific expression levels^53–59^. For example, many genes, even housekeeping genes, such as *OsTubA1* in rice^55^, *GhTUB1* in cotton^56^, α- and β-tubulin genes in *Populus*^57^ showed tissue-preferential expression. The *ACT1* gene showed different expression levels in the leaflet, flower, and young and mature fruits of blackberry and raspberry^58^. *PmAct1* and *PmAct2* showed distinct expression levels in different organs of beach plum^59^. In our analysis, the most stably expressed genes in the roots and leaves of ramie often differed even under the same growth conditions. Our results are consistent with those reported above and show that different ramie tissues under the same growth condition may require different internal reference genes during qPCR. However, to our knowledge, our study is among the first to demonstrate that different tissue x growth condition combinations may require different internal reference genes. Indeed, the suitable reference genes across the root and leaf samples of ramie plants under particular treatments as suggested by RefFinder were often inconsistent with those recommended separately for the roots or leaves. Taken together, our results indicate that there may not be a set of superior reference genes for all tissues of ramie, and that these suitable reference genes should be determined empirically according to experimental conditions and tissues.

Among the eight candidate genes screened in our study, most have a history of being used as internal reference genes for quantifying gene expressions through a variety of techniques. The relatively new one is *F-box*. *F-box* was recently shown to be the most stable reference gene under different experimental conditions in citrus fruits and in the common ivy (*Hedera helix*)^60,61^. In our study, we evaluated the *F-box* gene as a potential internal reference in ramie and found that it was among the most stably expressed in certain tissue x treatment combinations, including leaf samples of ramie plants subjected to *P*. *vexans* infection, BTH treatment, or Cd stresses and root samples of ramie plants subjected to SA treatment and cold or drought stresses. In contrast, *GAPDH*, a commonly used reference gene, performed relatively poorly under several experimental conditions, such as BTH, heat, cold, and salt stress treatments. Similar results have been reported for some other plants. For example, among those screened, *GAPDH* was the least suitable reference gene in purple false brome (*Brachypodium distachyon*), switchgrass (*Panicum virgatum*), qianhu (*Peucedanum praeruptorum*), and ryegrass (*Lolium multiflorum*) under abiotic stresses^11,45,53,62^.

### Conclusions and perspectives

In this study, we selected eight candidate genes based on ramie transcriptome data and from the common list of qPCR reference genes used in the plant kingdom to search for suitable reference genes for use in qPCR analysis under different conditions for ramie leaves and roots. Our analyses showed that each of the eight genes showed the highest ranking in at least one tissue x experimental treatment combination as suggested by at least one of the analytical programs. Among these eight genes, three (*ACT1*, *CYP2*, and *UBQ)* displayed relatively stable expression in the leaves under most experimental conditions, while *EF-1α*, *TUB*, and *ACT1* showed relatively stable expression in ramie roots under most experimental conditions. The most stable reference genes in leaf samples were often different from those in root samples, even under the same experimental conditions. We believe that the most stable reference genes screened in this study will improve the accuracy and standardization of investigations of ramie target gene expression under different stress conditions by qPCR analysis.

Note that the materials used in this study included only the roots and leaves of one ramie variety at the seedling stage. Even though we screened a diversity of biotic and abiotic stress conditions, the recommended reference genes identified here may not be the most suitable for other tissues (e.g. stems and flowers), other cultivars/varieties, and/or other developmental stages. In addition, although the eight genes selected here for analyses represent the commonly used reference genes across the plant kingdom and are among the most stably expressed based on transcriptome data, we cannot exclude the possibility that there might be more suitable reference genes in the ramie genome. Indeed, the variations in gene expressions observed here among the eight genes suggest that appropriate validations of candidate reference genes should be conducted for different tissues, genotypes, and developmental stages before a specific reference gene(s) is chosen for normalization of gene expression patterns.

## Supplementary information

Supplementary information

## Data Availability

We confirm that all the data associated with this manuscript are freely available and are presented either within the main manuscript file or in the Supplementary Materials section.
