# Supplementary material for: Selection of Reference Genes for qPCR Analyses of Gene Expression in Ramie Leaves and Roots across Eleven Abiotic/Biotic Treatments
Source: Sci Rep. 2019 Dec 27;9:20004. doi: 10.1038/s41598-019-56640-3 (PMC6934855; doi:10.1038/s41598-019-56640-3)
Supplement: Supplementary file 1 — Supplementary information [file 41598_2019_56640_MOESM1_ESM.pdf]

**Supplementary Information**

**Title of Manuscript: Selection of Reference Genes for qPCR Analyses of Gene Expression in Ramie Leaves and Roots across Eleven Abiotic/Biotic Treatments**

**Author List: Yongting Yu[1,†,♥], Gang Zhang[2,†], Yikun Chen[1], Qingqing Bai[2], Chunsheng Gao[1], Liangbin Zeng[1], Zhimin Li[1], Yi Cheng[1], Jia Chen[1], Xiangping Sun[1], Litao Guo[1], Jianping Xu[1,3*], Zhun Yan[1,†, ♥]**

[1] Department of Plant Protection, Institute of Bast Fiber Crops and Center for Southern Economic Crops, Chinese Academy of Agricultural Science, Changsha 410205, China;

[2] College of Pharmacy, Shaanxi University of Chinese Medicine, Xianyang 712406, China.

[3]Department of Biology, McMaster University, Hamilton, Ontario, L8S 4K1

*** Correspondence:**
Jianping Xu (jpxu@mcmaster.ca)

TEL: +86-731-88998585; Fax: +86-731-88998528
† These authors contributed equally to this work.

This Supplementary File contains the following information:

1. Figure S1. Melting curves of the eight candidate reference genes among biological and technical replicates.
2. Table S1. Digital expression analysis of candidate reference genes in ramie under various stresses.
3. Table S2. Information of eight candidate reference genes.
4. Table S3. Ranking the candidate reference genes across leaves and roots of ramie under different treatments as determined via RefFinder.

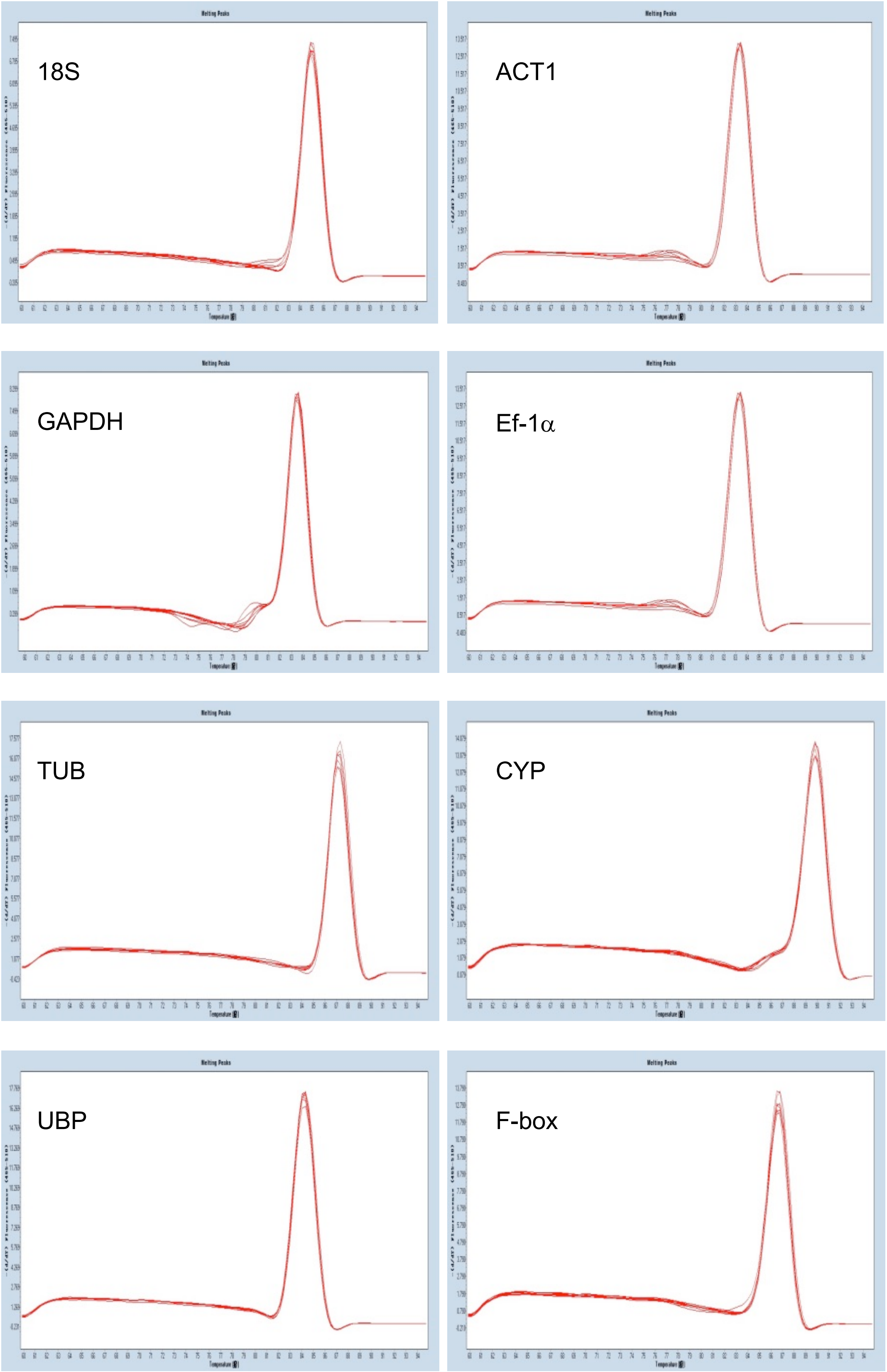


Figure S1. Melting curves of the eight candidate reference genes in ramie.

Table S1 Digital expression analysis of candidate reference genes in ramie under various stresses

| Gene Symbol | Gene name | Regulation under Nematode infection [1] | Regulation under Nematode infection [2] | Regulation under Cd stress [3] | Regulation under drought stress [4] | Regulation under ramie moth infestation [5] |
|---|---|---|---|---|---|---|
| *18S* | 18S rRNA (AF206870.1) | none | up | none | none | none |
| *ACT1* | Actin1 (DQ665832.2) | none | none | none | none | none |
| *GAPDH* | glyceraldehyde-3-phosphate dehydrogenase | none | none | none | none | down |
| *EF-1α* | Elongation factor 1-alpha | none | none | none | none | none |
| *αTUB* | a-tubulin | none | none | none | none | none |
| *CYP* | Cyclophilin2 | none | none | none | none | none |
| *UBQ* | Ubiquitin | none | none | none | none | none |
| *F-box* | F-box family | none | none | none | none | none |

Note: RNA-Seq based identification of differentially expressed genes in: stems and leaves (1, Zhu et al. 2014), roots (2, Yu et al. 2015) of ramie challenged by Pratylenchus coffeae, whole plants of ramie under CdCl2 stress (3, Liu et al. 2015), leaves and roots of ramie under draught stress (4, An et al. 2015), leaves of ramie infested by ramie moth larvae (5, Zeng et al. 2106).

References

1. Zhu, S., Tang, S., Tang, Q., & Liu, T. (2014). Genome-wide transcriptional changes of ramie (Boehmeria nivea L. Gaud) in response to root-lesion nematode infection. Gene, 552(1), 67-74.

2. Yu, Y., Zeng, L., Yan, Z., Liu, T., Sun, K., Zhu, T., & Zhu, A. (2015). Identification of ramie genes in response to Pratylenchus coffeae infection challenge by digital gene expression analysis. International journal of molecular sciences, 16(9), 21989-22007.

3.Liu, T., Zhu, S., Tang, Q., & Tang, S. (2015). Genome-wide transcriptomic profiling of ramie (Boehmeria nivea L. Gaud) in response to cadmium stress. Gene, 558(1), 131-137.

4. An, X., Chen, J., Zhang, J., Liao, Y., Dai, L., Wang, B., ... & Peng, D. (2015). Transcriptome profiling and identification of transcription factors in ramie (Boehmeria nivea L. Gaud) in response to PEG treatment, using illumina paired-end sequencing technology. International journal of molecular sciences, 16(2), 3493-3511.

5. Zeng, L., Shen, A., Chen, J., Yan, Z., Liu, T., Xue, Z., & Yu, Y. (2016). Transcriptome analysis of ramie (Boehmeria nivea L. Gaud.) in response to ramie moth (Cocytodes coerulea Guenée) infestation. BioMed research international, 2016.

Table S2 Information of eight candidate reference genes.

| Gene symbol | Gene description | Gene ID | Homolog ID | Gene expression | *E* score | Sequence identity (%) | Reference |
|---|---|---|---|---|---|---|---|
| *18S* | 18S rRNA | AF206870.1 | JF317363.1 | Debregeasia saeneb 18S ribosomal RNA gene | 0 | 99 | Zhang et al. 2011 |
| *ACT1* | Actin1 | DQ665832.2 | HQ163776 | Morus alba actin 1 (ACT1) mRNA, complete cds | 0 | 92 | Li et al. 2011 |
| *GAPDH* | glyceraldehyde-3-phosphate dehydrogenase | comp37700_c0 | XM_010093908 | Morus notabilis NADP-dependent glyceraldehyde-3-phosphate dehydrogenase (LOC21386841), mRNA | 0 | 88 | He et al. 2013 |
| *EF-1α* | Elongation factor 1-alpha | comp36076_c0 | JN399225.1 | Rosa multiflora elongation factor 1-alpha mRNA, complete cds | 0 | 91 | Klie & Debener. 2011 |
| *αTUB* | alpha-tubulin | comp24081_c0 | X67162 | P.amygdalus mRNA for alpha-tubulin | 3E-97 | 97 | Stocker et al. 1993 |
| *CYP2* | Cyclophilin2 | comp26478_c0 | NM_001357079 | Glycine max peptidyl-prolyl cis-trans isomerase CYP2 (GMCYP2), mRNA | 2E-180 | 90 | Mainali et al. 2014 |
| *UBQ* | Ubiquitin | comp20072_c0 | XM_016024811 | Ziziphus jujuba ubiquitin-60S ribosomal protein L40 (LOC107416326), mRNA | 4E-101 | 88 | Liu et al. 2014 |
| *F-box* | F-box family | comp30228_c0 | XM_024174059 | Morus notabilis putative F-box protein PP2-B12 (LOC21389887), mRNA | 3E-127 | 77 | He et al.2013 |

Table S3. Ranking the candidate reference genes across leaves and roots of ramie under different treatments as determined via RefFinder.

| Rank | Total | Abiotic | Hormone | *P. vexans* | SA | BTH | JA | ETH | GA3 | Heat | Cold | Na | Cd | PEG |
|---|---|---|---|---|---|---|---|---|---|---|---|---|---|---|
| 1 | ACT1<br>(1.19) | ACT1<br>(1.57) | GAPDH<br>(2.00) | F-box<br>(1.00) | F-box<br>(1.78) | F-box<br>(1.68) | 18S<br>(1.50) | F-box<br>(1.78) | 18S<br>(1.41) | 18S<br>(1.63) | F-box<br>(1.68) | ACT1<br>(1.19) | ACT1<br>(1.19) | EF-1a<br>(1.73) |
| 2 | GAPDH<br>(2.45) | UBQ<br>(2.34) | TUB<br>(2.45) | EF-1a<br>(1.86) | TUB<br>(2.06) | 18S<br>(1.97) | GAPDH<br>(2.21) | GAPDH<br>(2.91) | F-box<br>(1.86) | TUB<br>(2.21) | CYP2<br>(2.06) | F-box<br>(2.63) | F-box<br>(2.63) | GAPDH<br>(2.11) |
| 3 | TUB<br>(2.83) | F-box<br>(2.55) | ACT1<br>(3.13) | UBQ<br>(2.71) | EF-1a<br>(2.21) | EF-1a<br>(2.45) | ACT1<br>(2.28) | TUB<br>(3.13) | GAPDH<br>(3.66) | UBQ<br>(3.08) | UBQ<br>(2.51) | EF-1a<br>(2.99) | EF-1a<br>(2.99) | ACT1<br>(2.45) |
| 4 | F-box<br>(3.98) | EF-1a<br>(3.94) | CYP2<br>(3.87) | 18S<br>(4.00) | 18S<br>(4.40) | ACT1<br>(3.34) | TUB<br>(3.66) | UBQ<br>(3.64) | ACT1<br>(3.98) | F-box<br>(3.83) | TUB<br>(2.94) | CYP2<br>(4.05) | CYP2<br>(4.05) | F-box<br>(2.99) |
| 5 | 18S<br>(4.76) | 18S<br>(4.28) | F-box<br>(3.98) | ACT1<br>(5.23) | GAPDH<br>(4.43) | CYP2<br>(4.43) | EF-1a<br>(4.47) | EF-1a<br>(3.66) | EF-1a<br>(4.12) | ACT1<br>(3.94) | ACT1<br>(4.68) | TUB<br>(4.76) | TUB<br>(4.76) | 18S<br>(3.76) |
| 6 | UBQ<br>(5.48) | GAPDH<br>(4.47) | 18S<br>(4.33) | GAPDH<br>(6.19) | ACT1<br>(4.76) | TUB<br>(5.63) | F-box<br>(4.90) | CYP2<br>(4.45) | UBQ<br>(5.44) | CYP2<br>(4.16) | 18S<br>(6.24) | GAPDH<br>(4.79) | GAPDH<br>(4.79) | TUB<br>(6.00) |
| 7 | EF-1a<br>(6.19) | TUB<br>(6.24) | EF-1a<br>(4.45) | TUB<br>(6.70) | CYP2<br>(6.45) | GAPDH<br>(6.48) | UBQ<br>(7.00) | 18S<br>(5.24) | TUB<br>(5.66) | GAPDH<br>(6.09) | GAPDH<br>(6.26) | 18S<br>(5.42) | 18S<br>(5.42) | UBQ<br>(7.00) |
| 8 | CYP2<br>(6.40) | CYP2<br>(7.74) | UBQ<br>(7.44) | CYP2<br>(7.74) | UBQ<br>(7.00) | UBQ<br>(7.74) | CYP2<br>(8.00) | ACT1<br>(6.73) | CYP2<br>(6.96) | EF-1a<br>(8.00) | EF-1a<br>(7.24) | UBQ<br>(7.24) | UBQ<br>(7.24) | CYP2<br>(8.00) |
